# Supplementary material for: Quantifying the role of transcript levels in mediating DNA methylation effects on complex traits and diseases
Source: Nat Commun. 2022 Dec 7;13:7559. doi: 10.1038/s41467-022-35196-3 (PMC9729239; doi:10.1038/s41467-022-35196-3)
Supplement: Supplementary file 1 — Supplementary Information [file 41467_2022_35196_MOESM1_ESM.pdf]

Supplementary Materials for

Quantifying the role of transcript levels in mediating DNA methylation effects on complex traits and diseases

Marie C. Sadler[1,2,3,*], Chiara Auwerx[1,2,3,4], Kaido Lepik[1,2,3], Eleonora Porcu[1,2,3,4,5], Zoltán Kutalik[1,2,3,5,*]

[1] University Center for Primary Care and Public Health, Lausanne, Switzerland

[2] Department of Computational Biology, University of Lausanne, Lausanne, Switzerland

[3] Swiss Institute of Bioinformatics, Lausanne, Switzerland

[4] Center for Integrative Genomics, University of Lausanne, Lausanne, Switzerland

[5]Authors jointly supervised this work

*Corresponding authors: marie.sadler@unil.ch, zoltan.kutalik@unil.ch

Table of Contents

Table of Contents *2*

Supplementary Figures *3*

3S-MVMR Workflow **3**

MR parameter sensitivity analyses **4**

Simulation studies **6**

Mediation proportions by physiological and structural categories **10**

Mediation through the top transcript mediator **11**

MVMR sensitivity analyses **12**

Stratification by DNAm annotations **19**

Mediation analyses with uncorrelated mediators **20**

Multi-omics mechanisms of action **22**

Supplementary Tables *24*

Supplementary Figures

3S-MVMR Workflow

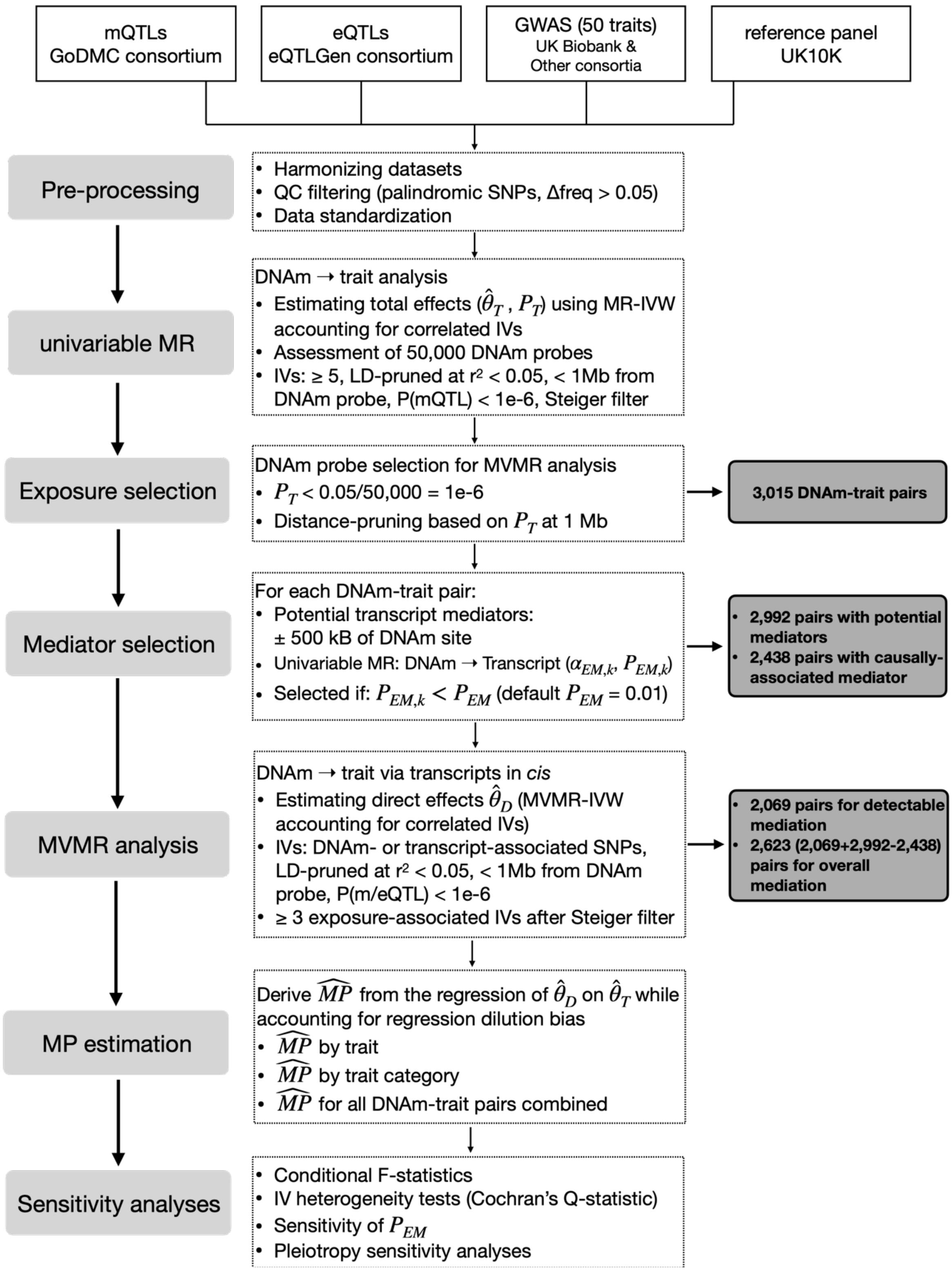

Supplementary Figure 1. **3S-MVMR workflow**. DNAm-to-trait mediation analysis workflow.

MR parameter sensitivity analyses

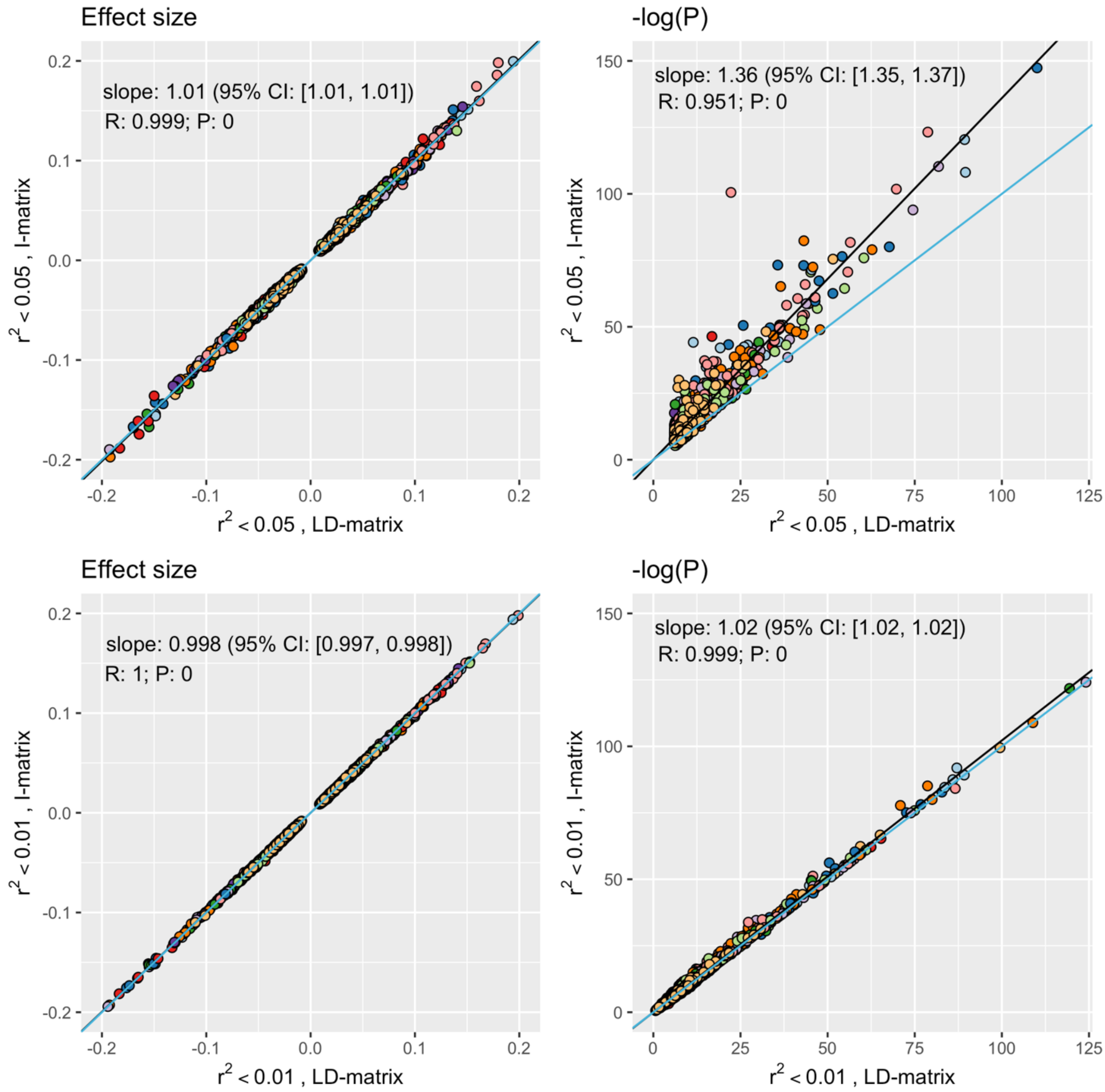

Supplementary Figure 2. **Sensitivity analyses to assess the influence of the LD-matrix**. The analyses were performed on the 3,015 DNAm-trait pairs for which there was a significant causal effect (Supplementary Fig. 1). Effect sizes and corresponding p-values were derived from IVW-MR estimates, once accounting for correlation between instruments (i.e. inclusion of LD-matrix) and once setting the LD-matrix to the identity matrix I (i.e. standard IVW estimates). The first row shows the results when setting the pruning threshold r^2 to 0.05 and the second row to 0.01. The analyses show that MR effect estimates are not affected by the LD-matrix, but if the LD-matrix was omitted p-values of causal effects were deviating towards lower values at $r^2 = 0.05$, while this was no longer the case at $r^2 = 0.01$. This indicates that at $r^2 = 0.01$, independence between IVs can be confidently claimed, while at $r^2 > 0.01$, the LD-matrix should be included to avoid false positives. Each dot represents a DNAm-trait pair colour-coded by the physiological category of the trait as defined in Supplementary Fig. 8. The slope is indicated in black with numerical values shown in the plotting area and the identity line in blue. The reported p-values (*P*) for the corresponding Pearson correlations (*R*) arise from a two-sided t-test and are shown in the plotting area.

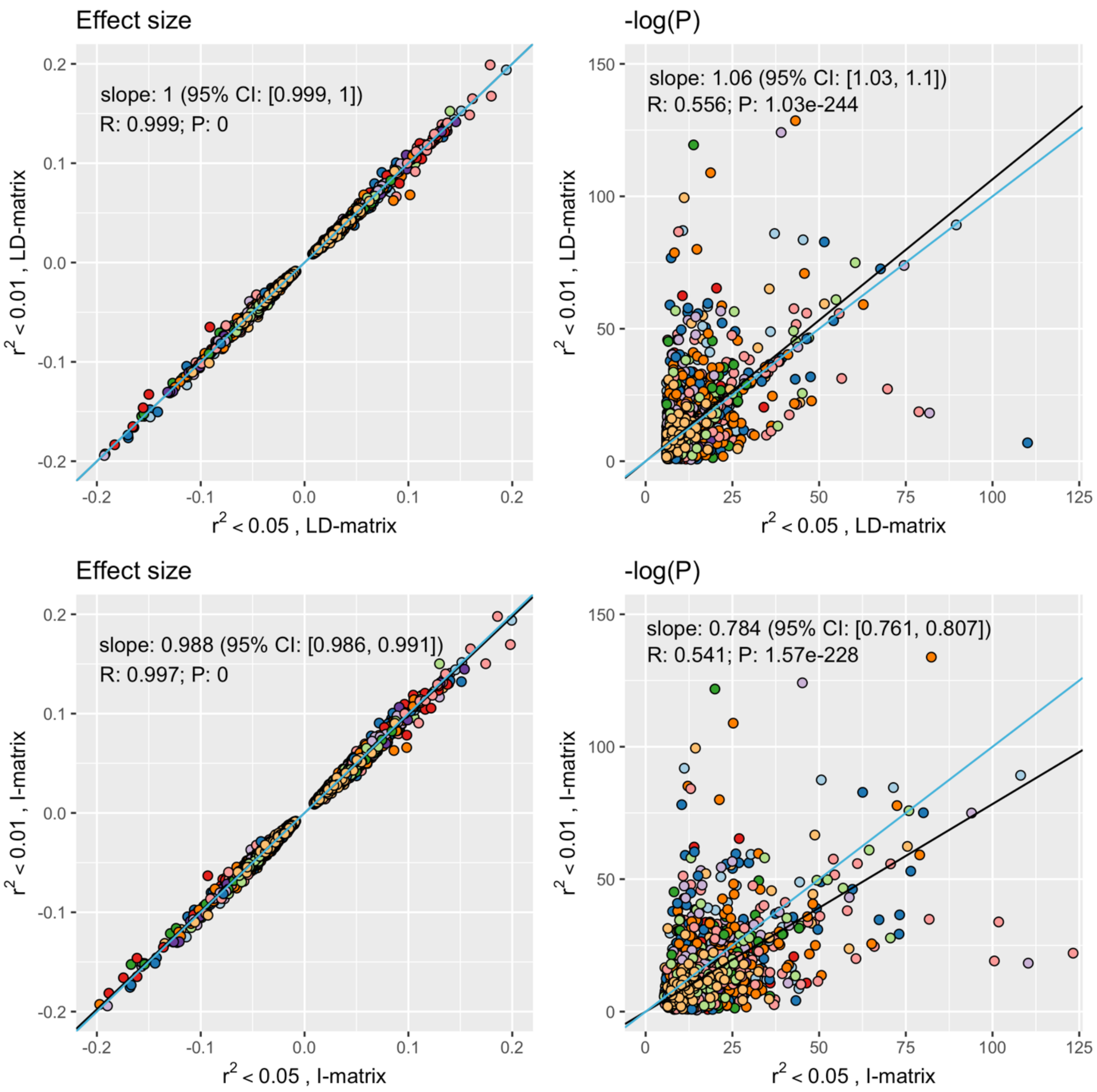

Supplementary Figure 3. Sensitivity analyses to assess the influence of the pruning threshold r^2. Again, the analyses were performed on the 3,015 DNAm-trait pairs for which there was a significant causal effect (Supplementary Fig. 1). Effect sizes and corresponding p-values were derived from IVW-MR estimates at different pruning thresholds. The first row shows the results when including the LD-matrix and the second row when setting it to the identity matrix I. The analyses show that MR effect estimates are not affected by the different pruning thresholds. However, the significance levels (p-values) can differ between both thresholds, although no overall significant difference was found as shown by the slope (provided the LD-matrix is included). When omitting the LD-matrix, lower p-values were found at $r^2 = 0.05$, a threshold at which, however, neglecting correlation between IVs was demonstrated to be an invalid approach (Supplementary Fig. 2). Each dot represents a DNAm-trait pair colour-coded by the physiological category of the trait as defined in Supplementary Fig. 8. The slope is indicated in black with numerical values shown in the plotting area and the identity line in blue. The reported p-values (*P*) for the corresponding Pearson correlations (*R*) arise from a two-sided t-test and are shown in the plotting area.

Simulation studies

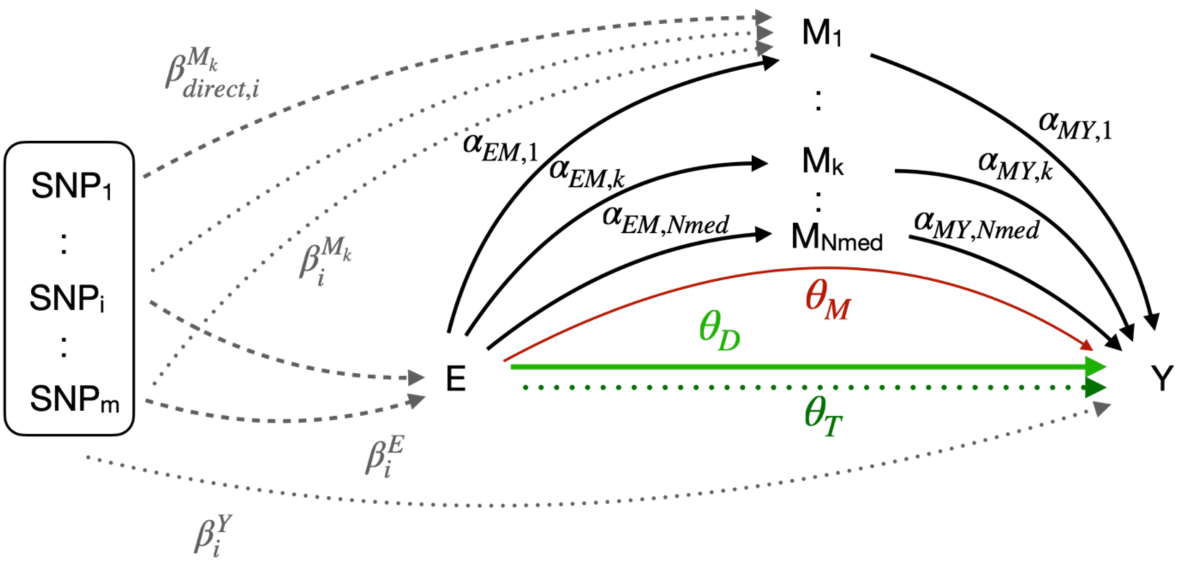

Supplementary Figure 4. **Model used in the simulation settings to estimate the total, direct and indirect causal effects ($\boldsymbol{\Theta_T}$, $\boldsymbol{\Theta_D}$ and $\boldsymbol{\Theta_M}$, respectively).** Genetic variants (SNPs) are either directly associated (dashed arrow) with the exposure *E* or mediators M_k (1 to N_{med}), or indirectly (dotted arrow) with M_k through *E*. The genetic effect sizes are denoted by β, where β^E are direct effects to *E*, β^{M_k} total effects to M_k made of the direct effects $\beta^{M_k}_{direct}$ to M_k and the indirect effects through *E*, and β^Y are total effects to the outcome *Y* through either *E* or *M*. Causal effects from *E* to M_k are denoted by $\alpha_{EM,k}$ and causal effects from *M* to *Y* by $\alpha_{MY,k}$.

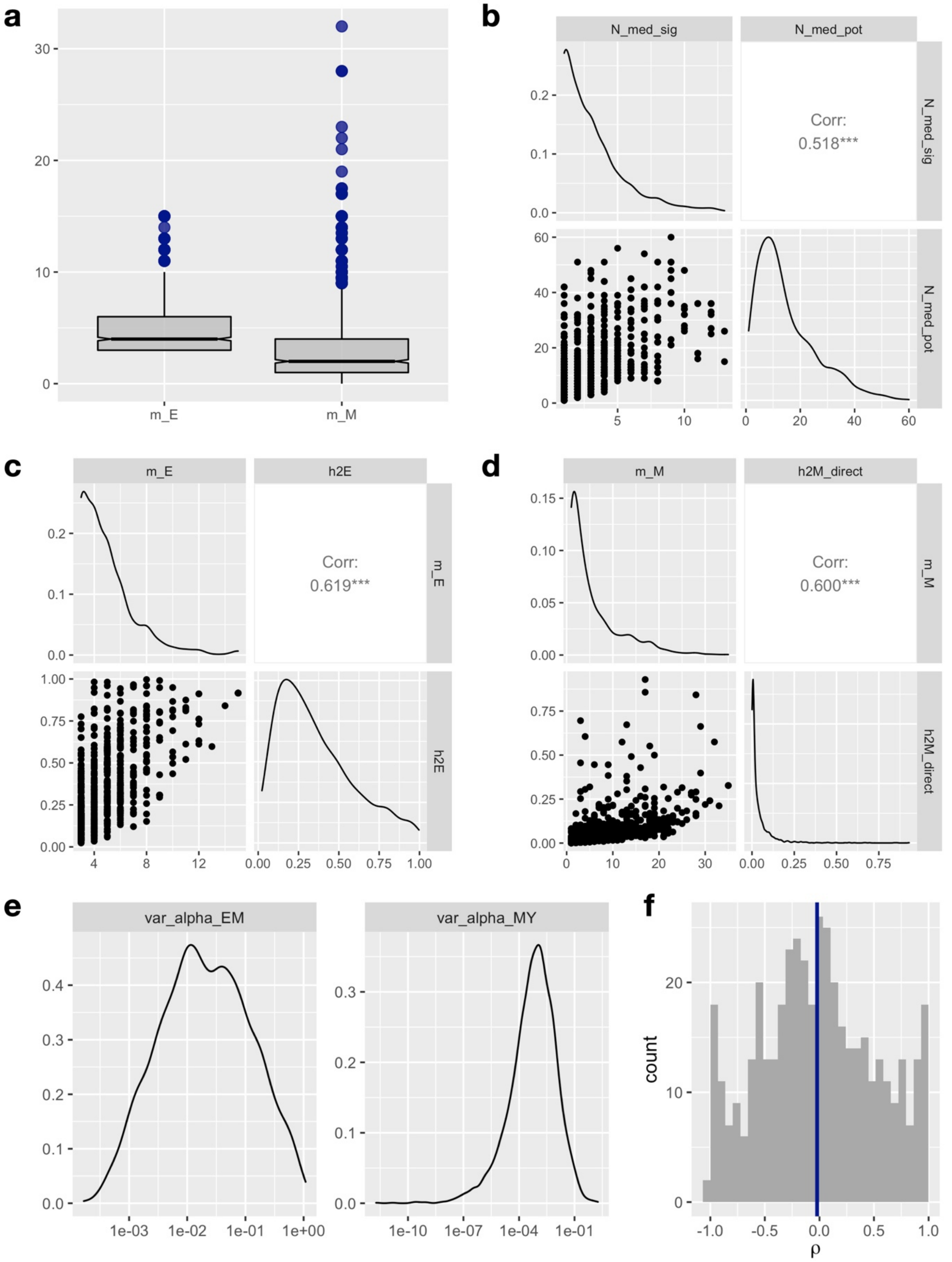

Supplementary Figure 5. Distribution of the simulation parameters as observed in real data. Numerical values (interquartile ranges, mean) are shown in Supplementary Table 1 and parameter choices of the different simulation settings to explore the full range of realistic parameter estimates are summarized in Supplementary Table 2. **a** Distribution of the number of exposure-associated (m_E) and mediator-associated (m_M) independent instrumental variables (IVs) calculated on 1,836 DNAm-trait pairs. Boxes bound the 25th, 50th (median, centre), and the 75th quantile. Whiskers range from minima (Q1 – 1.5*IQR) to maxima (Q3 + 1.5*IQR) with points above or below representing potential

outliers. **b** Distribution of the number of selected mediators ($N_{\text{med,sig}}$) and of the total number of potential mediators in the region ($N_{\text{med,pot}}$ with $N_{\text{med,pot}} \geq N_{\text{med,sig}}$). The Pearson correlation coefficient (Corr) is shown with the stars (***) indicating that the corresponding p-value (two-sided test-statistic) was below 2.2e-16 (exact p-value equalled 4.14e-64). **c** Distribution of the exposure heritability (h_E^2) in relationship with the number of exposure-associated IVs (m_E). Same Pearson correlation calculation as in b (exact p-value equalled 3.12e-93). **d** Distribution of the direct heritability of each mediator k ($h^2_{M,direct,k}$) - ignoring the heritability coming through the exposure - in relationship with the number of mediator-associated IVs (m_M). Same Pearson correlation calculation as in b (exact p-value equalled 5.08e-176). **e** Distribution of the variance (across all mediators) of the exposure-to-mediator causal effects (var($\alpha_{EM,k}$)) and mediator-to-outcome effects (var($\alpha_{MY,k}$)) as estimated by $\widehat{\alpha}_k^2 - \text{se}(\widehat{\alpha}_k)^2$. **f** Distribution of the correlation (ρ) between $\alpha_{EM,k}$ and $\alpha_{MY,k}$. Estimation was done by considering DNAm-trait pairs with at least 3 mediators and calculating for each pair the correlation between $\alpha_{EM,k}$ and $\alpha_{MY,k}$ that were estimated for each mediator.

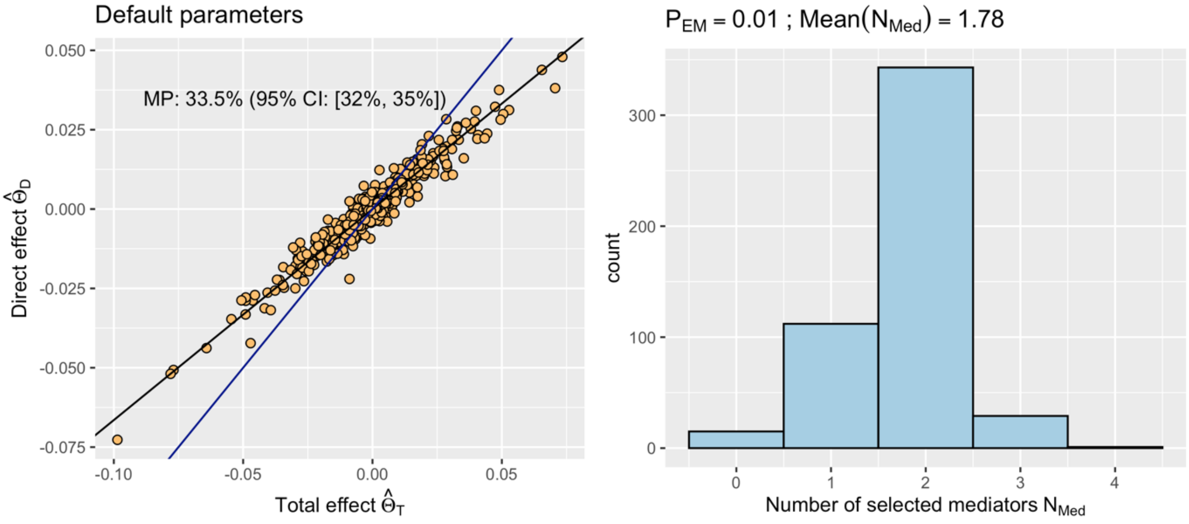

Supplementary Figure 6. Simulation results with the parameter default settings as indicated in Supplementary Table 2. 500 exposure-outcome pairs were simulated and for each a direct and total effect was estimated. The estimated mediation proportion (%) together with the 95% CI are displayed in the plot area (resulting from the regression of $\widehat{\Theta}_D$ against $\widehat{\Theta}_T$) with the corresponding slope plotted in black (blue line represents the identity line). Mediators were selected based on a p-value threshold P_{EM} and the distribution of the selected number of mediators (among a set of 12 potential mediators) is shown in the histogram. The true number of relevant mediators was 2 and the true MP was 35% (Supplementary Table 2).

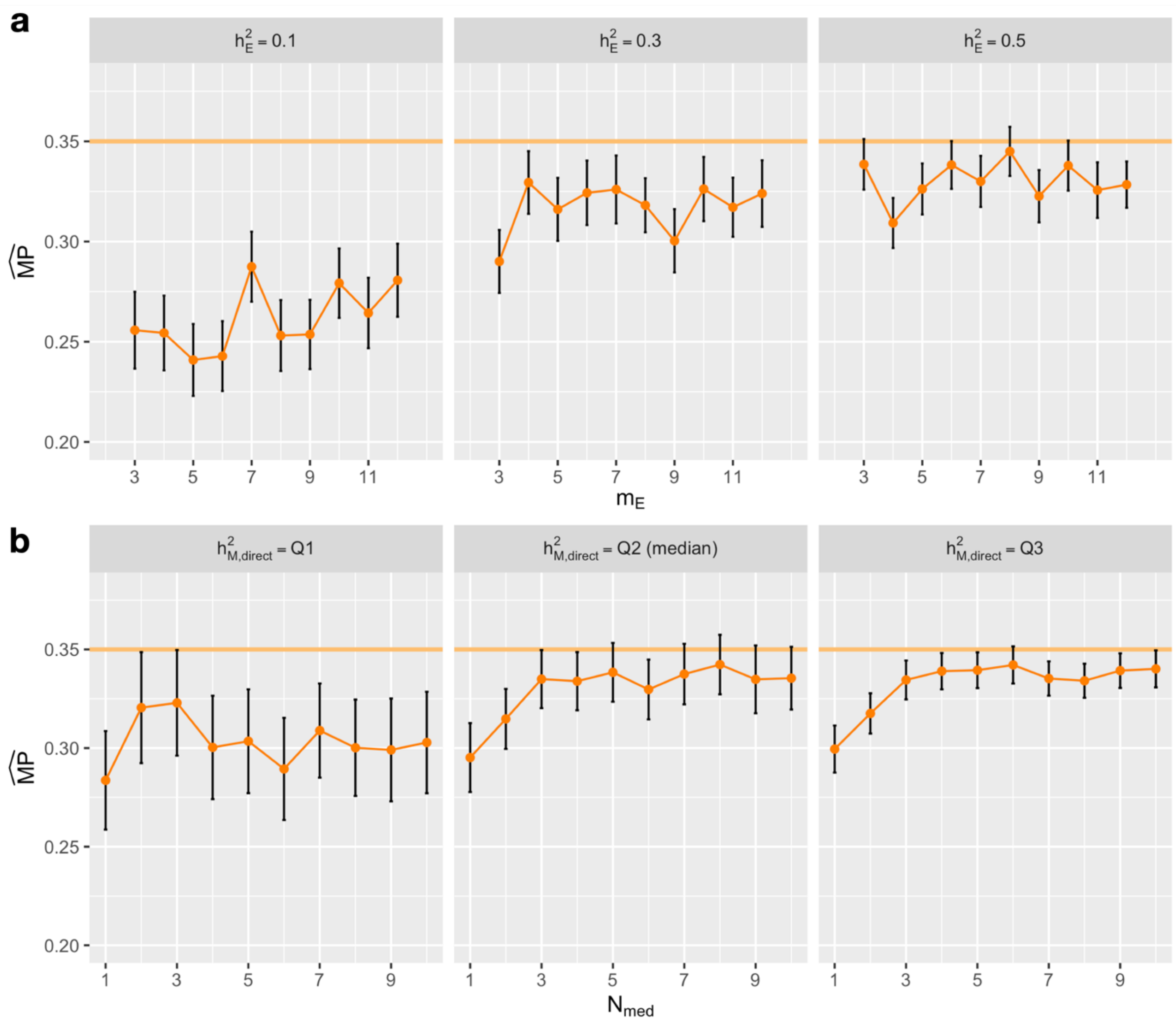

Supplementary Figure 7. Simulation results varying m_E and N_{med} (Supplementary Table 2). a The number of exposure-associated instrumental variables (IVs) m_E was changed for different exposure heritabilities h_E^2. The estimated mediation proportions were more dependent on h_E^2 than on the polygenicity of the exposure. **b** Dependence of the estimated mediation proportion on the number of true mediators N_{med} (i.e., mediators contributing to the indirect effect) stratified by $h^2_{M,direct}$. Underestimations were observed for fewer mediators (1-2) and when the direct mediator heritability was low (first quartile). With fewer true mediators N_{med}, missing a relevant mediator has a greater impact on the estimated mediation proportion than if multiple N_{med} are contributing towards the mediated effect. Error bars represent 95% CI calculated on 500 simulated exposure-outcome pairs.

Mediation proportions by physiological and structural categories

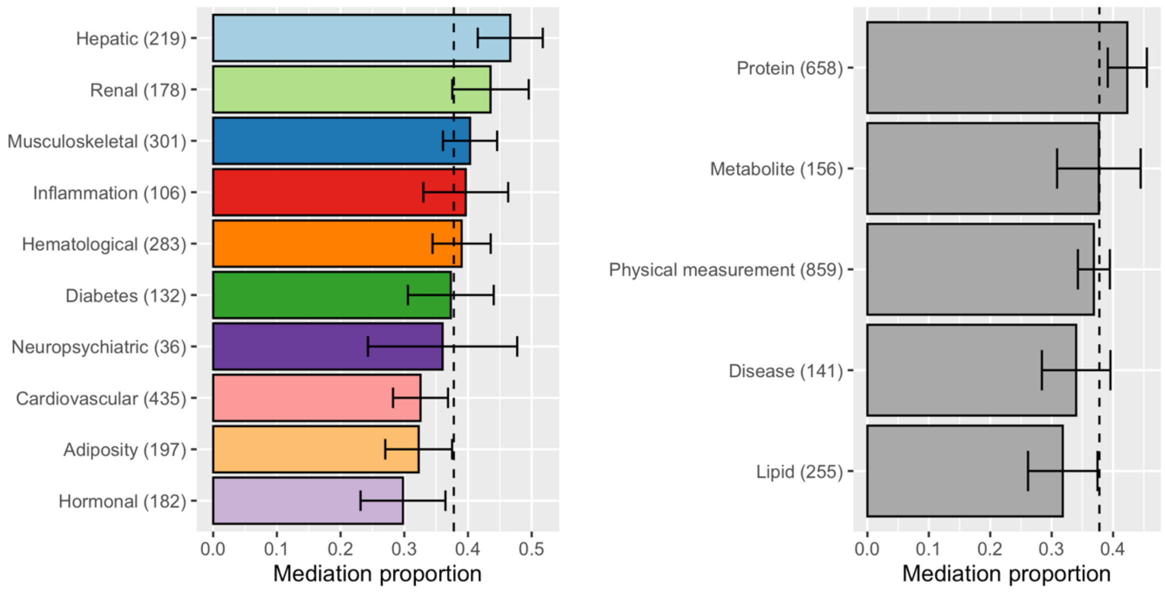

Supplementary Figure 8. Mediation proportion of traits grouped by physiological (left) and structural (right) categories. Further information about trait classification are shown in Supplementary Data 1. The vertical dotted lines denote the mean mediation proportion across all DNAm-trait pairs. 95% confidence intervals are represented by the error bars. $\widehat{\mathrm{MP}}s$ per category were derived by regressing $\hat{\theta}_D$ against $\hat{\theta}_T$. The number of DNAm-trait pairs falling into each category and on which the regression was performed is indicated in parentheses.

Mediation through the top transcript mediator

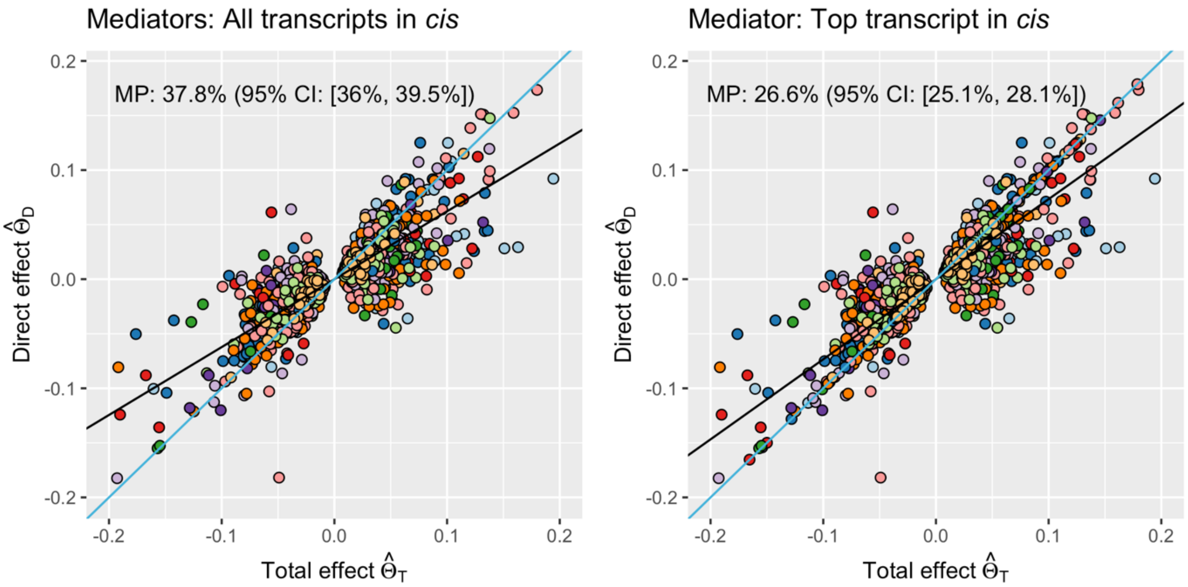

Supplementary Figure 9. Mediation through the top mediator. When restricting the mediation through the top transcript, i.e., the transcript most significantly associated to the DNAm site, the mean mediation proportion drops from 37.8% to 26.6% (evaluated are the 2,069 pairs with at least 1 causally associated transcript in *cis*). Plotted is the direct effect against the total effect together with the slope (black line). Each dot represents a DNAm-trait pair colour-coded by the physiological category of the trait as defined in Supplementary Fig. 8. The identity line is plotted in blue.

MVMR sensitivity analyses

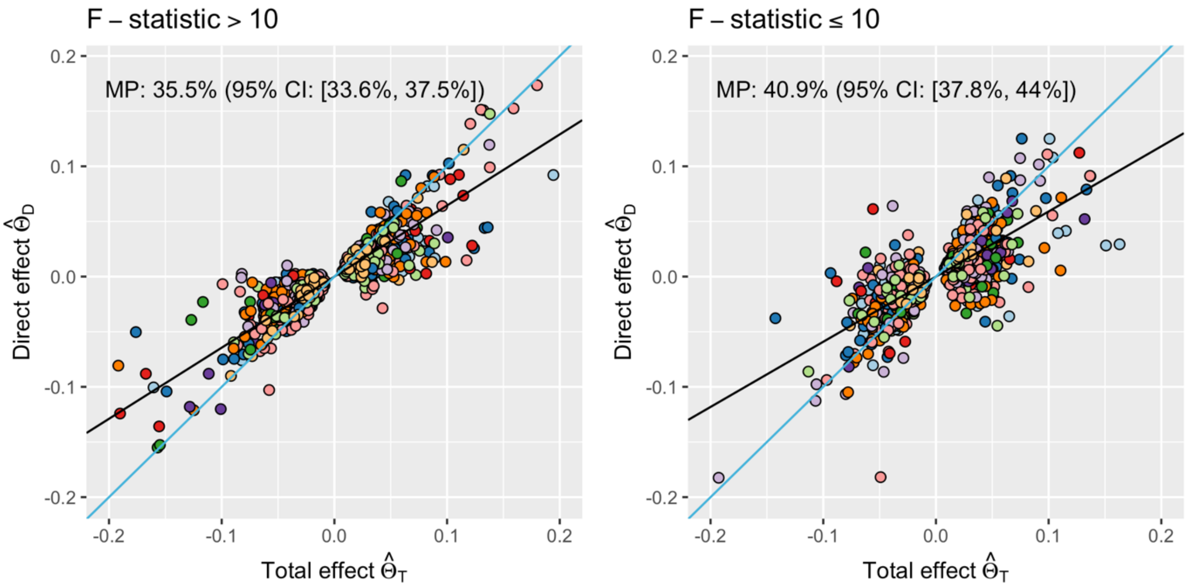

Supplementary Figure 10. MVMR sensitivity analysis to stratify DNAm-trait pairs by their conditional F-statistic. At an F-statistic below 10, the mediation analysis might suffer from weak instrument bias which can result in unreliable direct effect estimates. Among the 2,069 DNAm-trait pairs, 1,061 had an F-statistic above 10 and 1,008 below. DNAm-trait pairs are colour-coded by the physiological category of the trait as defined in Supplementary Fig. 8. The slope is plotted in black (numerical values shown in plotting area resulting from the regression of $\hat{\Theta}_D$ against $\hat{\Theta}_T$) and the identity line in blue.

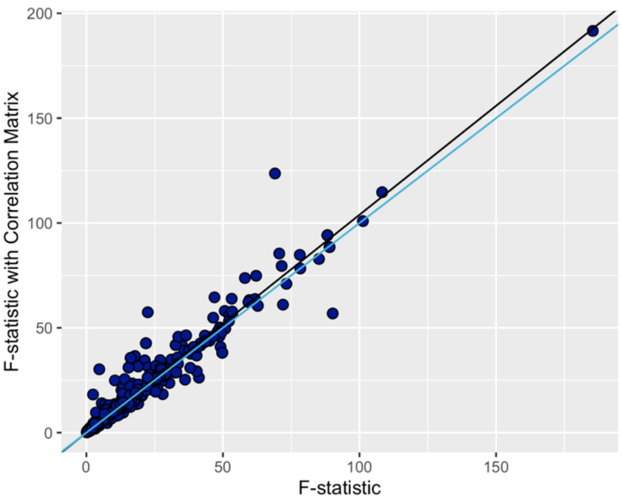

Supplementary Figure 11. Conditional F-statistics with and without the correlation matrix between mediators. Conditional F-statistics with transcript-transcript correlations were calculated for all DNAm-trait pairs with at least 2 mediators and for which at least half of them had available correlation data. In total, 1,208 pair were assessed with the mean F-statistics being 13.55 with the correlation matrix and 12.85 without. The slope is plotted in black and the identity line in blue.

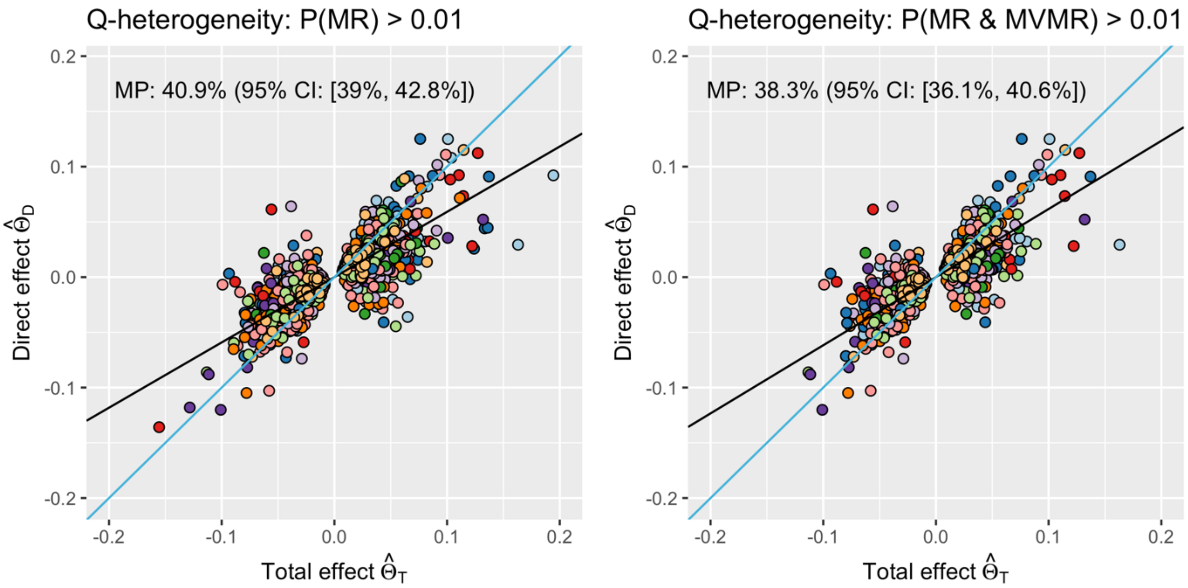

Supplementary Figure 12. MVMR sensitivity analysis to test for heterogeneity within the IV set. In the left figure, the 2,069 were filtered for those that showed no signs of heterogeneity in the univariable MR analyses (Q-heterogeneity p-value > 0.01; 1,757 pairs). In the right figure, the filtering was applied on the p-values of the Q-statistics of both the univariable and multivariable MR analyses (1,405 pairs). DNAm-trait pairs are colour-coded by the physiological category of the trait as defined in Supplementary Fig. 8. The slope is plotted in black (numerical values shown in plotting area resulting from the regression of $\hat{\Theta}_D$ against $\hat{\Theta}_T$) and the identity line in blue.

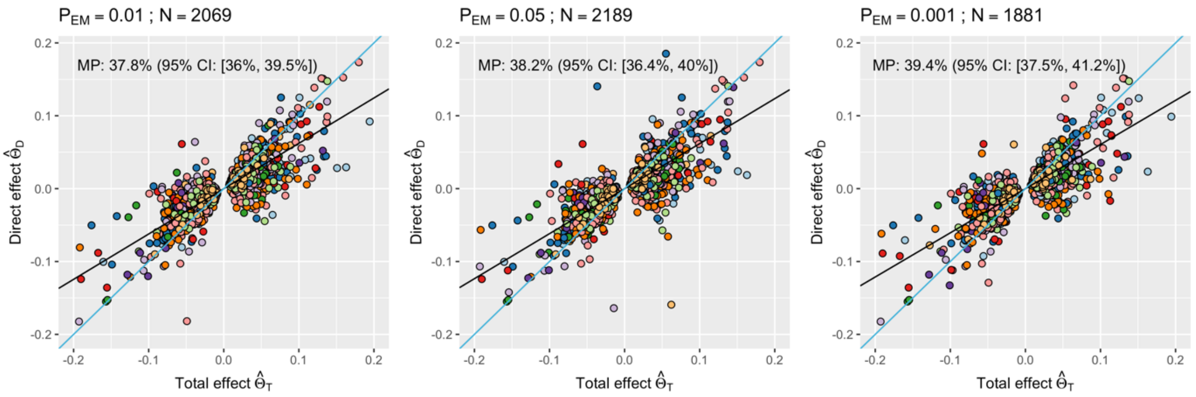

Supplementary Figure 13. MVMR sensitivity analysis to assess the influence of the P_{EM} thresholds to select mediators in case of "detectable mediation" analyses. Shown are the results for three thresholds (0.01, 0.05 and 0.001 from left to right). The calculation of the MP is done on DNAm-trait pairs (*N* pairs) with at least 1 transcript in the *cis* region causally associated to the DNAm site. DNAm-trait pairs are colour-coded by the physiological category of the trait as defined in Supplemetary Fig. 8. The slope is plotted in black (numerical values shown in plotting area resulting from the regression of $\hat{\Theta}_D$ against $\hat{\Theta}_T$) and the identity line in blue.

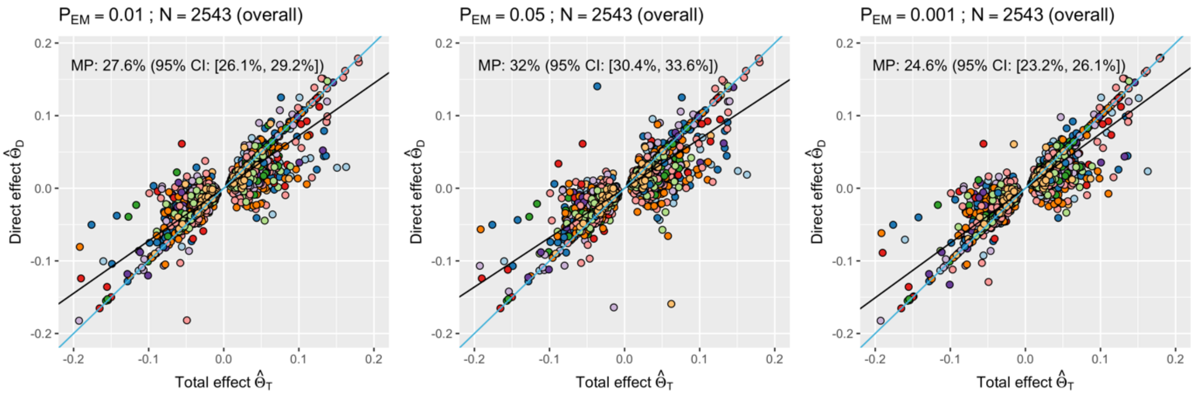

Supplementary Figure 14. MVMR sensitivity analysis to assess the influence of the P_{EM} thresholds to select mediators in case of the overall MP. Shown are the results for three thresholds (0.01, 0.05 and 0.001 from left to right). The overall MP is calculated on all DNAm-trait pairs (*N* pairs) with least 1 transcript in the *cis* region (not necessarily causally associated to the exposure) and for which a mediation analysis could be performed in all three settings (the number of IVs being the limiting factor). DNAm-trait pairs are colour-coded by the physiological category of the trait as defined in Supplementary Fig. 8. The slope is plotted in black (numerical values shown in plotting area resulting from the regression of $\hat{\Theta}_D$ against $\hat{\Theta}_T$) and the identity line in blue.

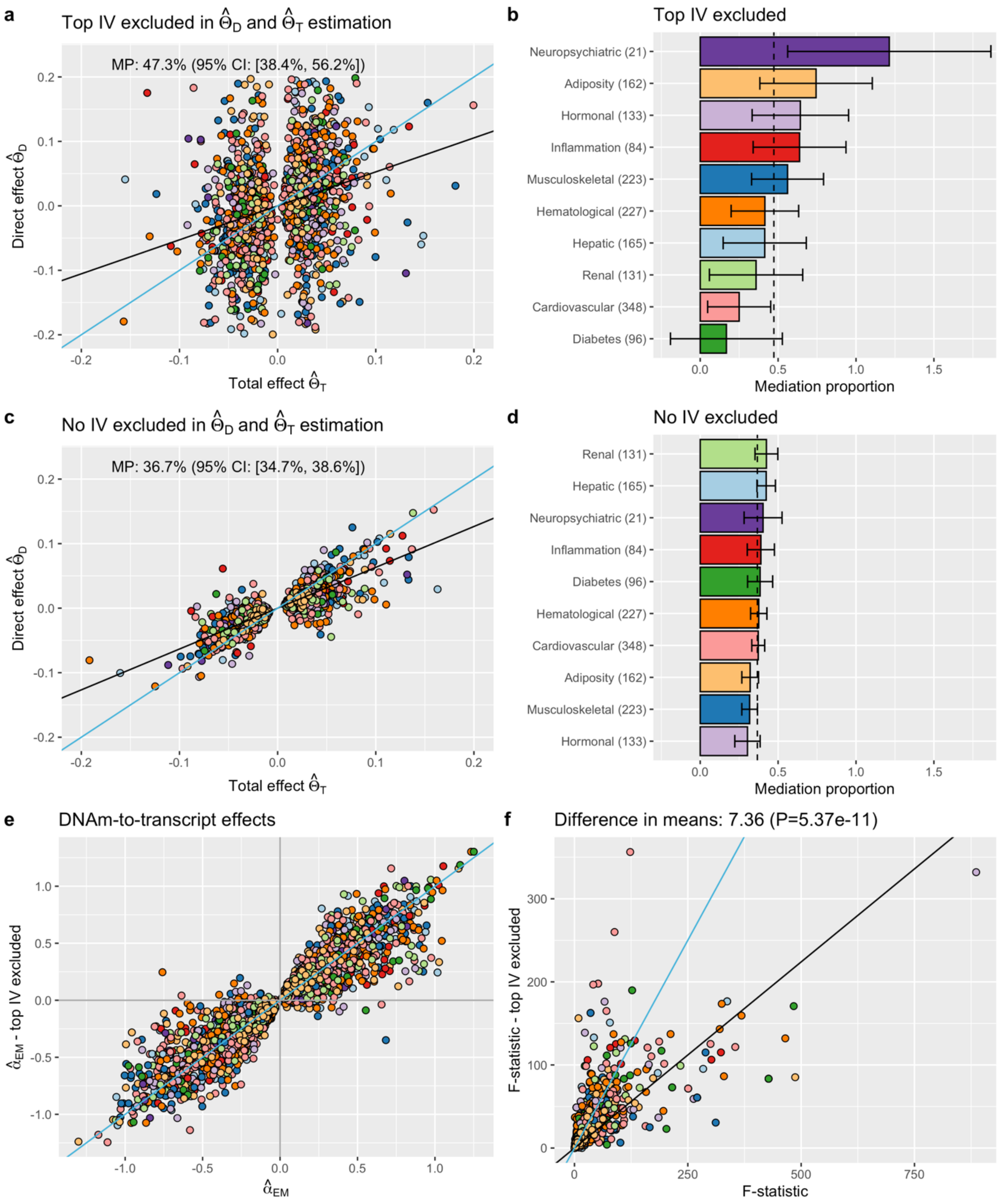

Supplementary Figure 15. MVMR sensitivity analysis excluding the top instrumental variable (pleiotropy sensitivity analysis). Mediation analyses were conducted for all DNAm-trait pairs with at least 3 exposure-associated IVs after excluding the top IV (i.e., exposure-associated IV with the lowest p-value; 1,590 DNAm-trait pairs). **a** $\widehat{\text{MP}}$ and 95% CI calculated on these pairs excluding the top IV in both the total and direct effect calculation. The slope is shown by the black line and the identity line by the blue line. **b** Corresponding MPs of traits grouped by physiological categories. The vertical dotted line corresponds to the mean MP across all DNAm-trait pairs and error bars represent the 95% CI. $\widehat{\text{MP}}$s per category were derived by regressing $\hat{\Theta}_D$ against $\hat{\Theta}_T$. **c**, **d** Same analysis as in **a** and **b**, respectively, but without excluding the top IV (same 1,590 pairs). **e** DNAm-to-transcript MR effects (α_{EM}) of the exposure-mediator pairs included in the mediation analyses of the 1,590 DNAm-trait pairs are shown before and after the exclusion of the top IV. **f** Conditional F-statistics calculated on the 1,590 DNAm-

trait pairs before and after the exclusion of the top IV. Conditional F-statistics were on average 7.36 higher before excluding the top IV (two-sided t-test p-value = 5.37e-11) pointing out that weak instrument bias was more present in the analyses where the top IV was missing.

Overall, the analyses show that excluding the top IV results in noisier MR estimates as a consequence of weaker instruments. While the top IV is crucial in getting robust molecular MR estimates, the analyses show that the remaining IVs support same effect size magnitudes and directionalities as the top IV.

Specifically, excluding the top IV significantly increased the MP estimated over all the DNAm-trait pairs (panel **a** vs **c**, P_{diff} = 0.0228). This difference is likely due to weak instrument bias as it was not present for pairs with F>10 (845/1,590 pairs, MP = 40.9%, 95% CI: [29.3%, 52.4%] – top IV excluded; P_{diff} = 0.48). The estimated MP did not depend on the conditional F-statistic when all IVs were considered (Supplementary Fig. 10).

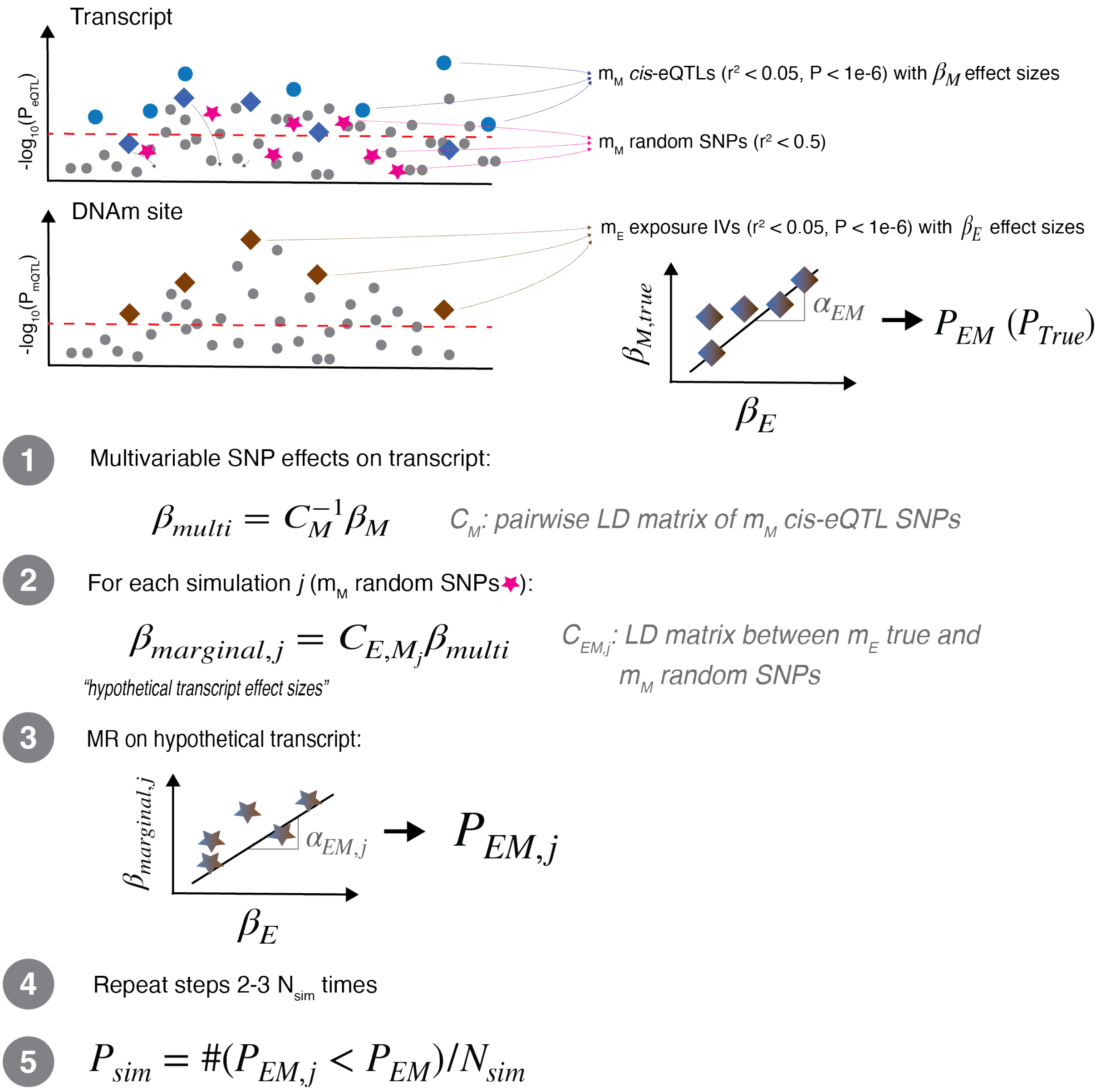

Supplementary Figure 16. Schematic illustrating the horizontal pleiotropy simulation analysis to assess the possibility of DNAm-to-transcript associations because of horizontal pleiotropy as a result of LD between mQTLs and eQTLs. First DNAm-transcript pairs with a significant MR effect at $P_{EM} < 1e$-6 are selected. Then, multivariable SNP effects on the transcript are calculated based on independent *cis*-eQTLs (step 1). In each of the following simulations, m_M random SNPs are selected for which marginal SNP-transcript effects are calculated. Note that these hypothetical transcript effect sizes have identical multivariable eQTL effect size distribution as the real transcript (step 2). Next, a univariable MR analysis on this hypothetical transcript yields $P_{EM,j}$ (step 3). Steps 2-3 are repeated N_{sim} times (step 4) which allows to calculate the simulation p-value P_{sim} (step 5).

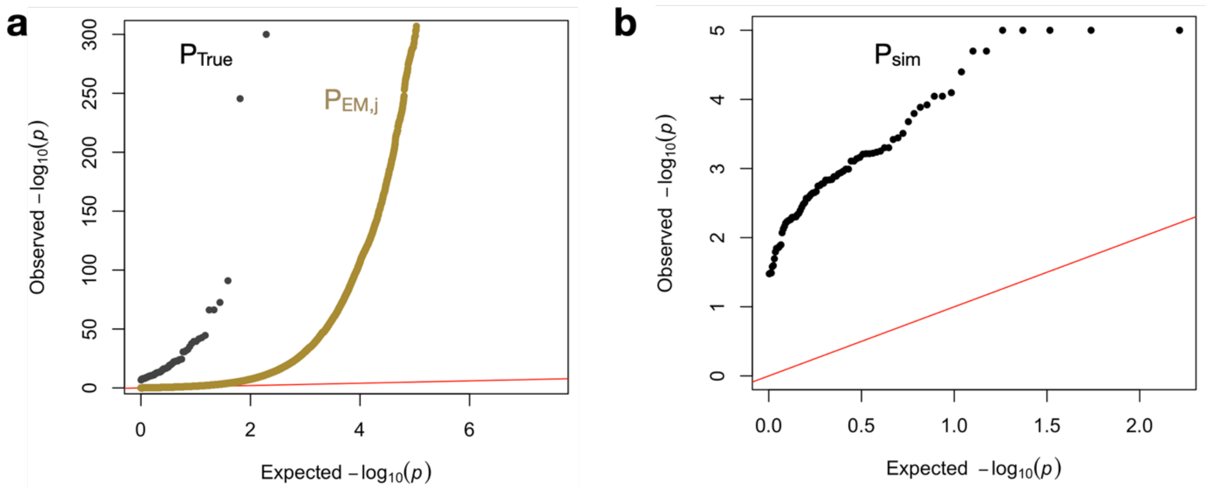

Supplementary Figure 17. Simulation analysis to assess the possibility of DNAm-to-transcript associations due to horizontal pleiotropy. For a significant DNAm-to-transcript MR association (P_{EM}, herein called P_{True}), we performed simulation tests (N_{sim} = 100,000) by randomly selecting eQTL-SNPs with identical multivariable eQTL effects in the region (Supplementary Fig. 16). Each simulated marginal eQTL effect estimate resulted in a random DNAm-to-transcript MR estimate ($P_{EM,j}$) from which we could derive the simulation p-value ($P_{sim} = \#(P_{EM,j} < P_{True})/N_{sim}$). **a** Comparison (normal QQ-plot) of the true (P_{True}) and random ($P_{EM,j}$) p-values from 100 DNAm-transcript MR estimates (the transcript outcome being the true and hypothetical transcript *j* from each simulation run, respectively). **b** Normal QQ-plot of the simulation p-values P_{sim}.

The analysis shows that while MR p-values from hypothetical transcript effects are inflated, they are much less significant than the true p-values ensuring that horizontal pleiotropy is not at the root of observed methylation-expression causal effects.

Stratification by DNAm annotations

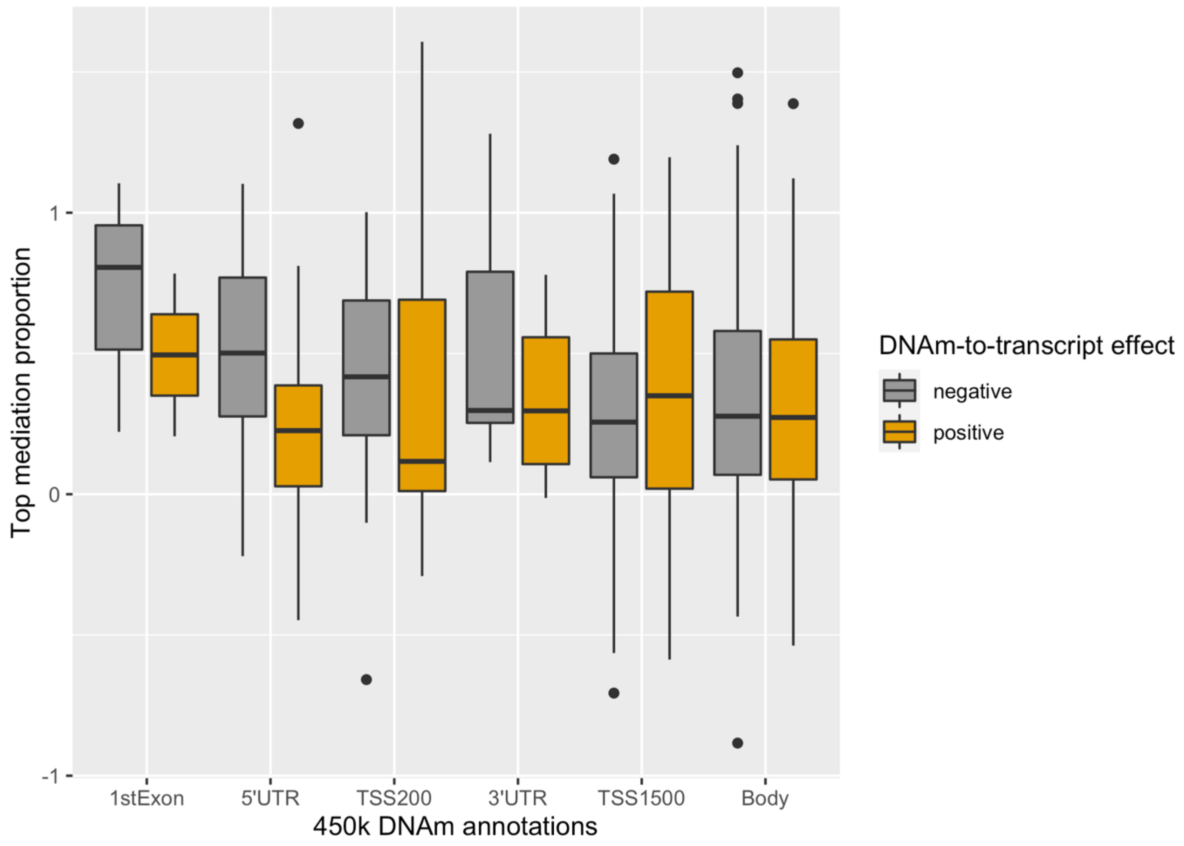

Supplementary Figure 18. Mediation proportion stratified by DNAm site location. Boxplots representing the top mediation proportion (MP_{top}) stratified by DNAm site location with respect to the top mediator and by the causal effect direction of the DNAm on the transcript level. The annotation groups are shown in decreasing order with respect to the mediation proportion (negative and positive DNAm-to-transcript effect pairs combined). Number of DNAm-trait pairs within each boxplot are as follows: 1stExon (negative: 3, positive: 2), 5'UTR (negative: 60, positive: 26), TSS200 (negative: 33, positive: 15), 3'UTR (negative: 14, positive: 12), TSS1500 (negative: 68, positive: 66), Body (negative: 172, positive: 104). Boxes bound the 25th, 50th (median, centre), and the 75th quantile. Whiskers range from minima (Q1 – 1.5*IQR) to maxima (Q3 + 1.5*IQR) with points above or below representing potential outliers. Note that annotations were not available for all DNAm sites and DNAm sites mapping to multiple annotations were omitted.

Mediation analyses with uncorrelated mediators

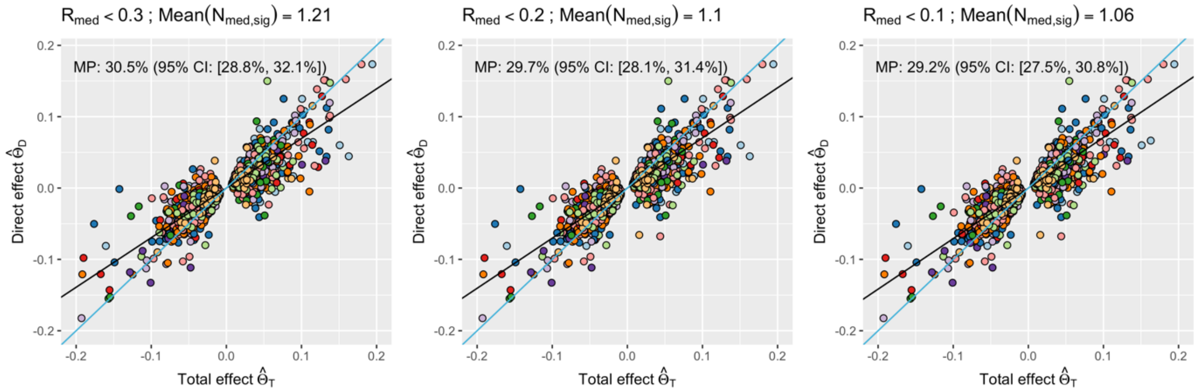

Supplementary Figure 19. Mediation analysis with uncorrelated mediators. Mediation analyses conducted with uncorrelated mediators at different R_{med} thresholds (0.3, 0.2, and 0.1 from left to right) for all 2,069 DNAm-trait pairs (colour-coded by the physiological category of the trait as defined in Supplementary Fig. 8). R_{med} is the maximum correlation between the mediators for a given exposure-outcome pair. As this threshold decreases, the average number of selected mediators ($N_{med,sig}$) decreases. The slope (black line) and the mediation proportion together with the 95% CI are displayed in the plot area (blue line represents the identity line).

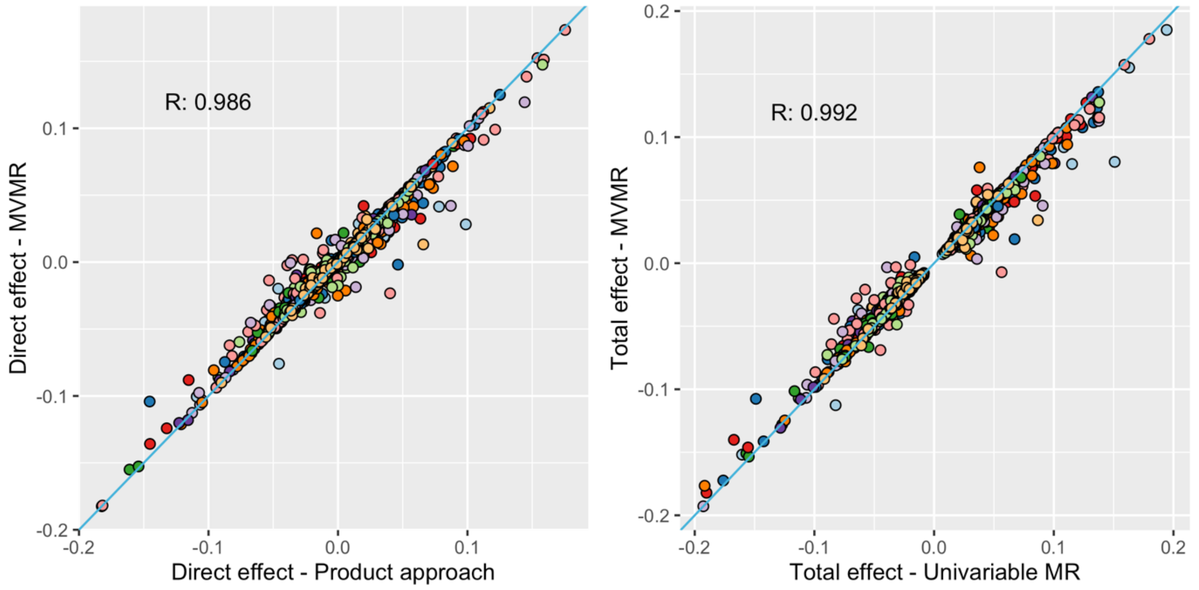

Supplementary Figure 20. Agreement between the product of coefficients and difference in coefficients methods to estimate direct and indirect effects. In the left panel, the agreement between direct effects estimated from the multivariable Mendelian randomization regression (MVMR, difference in coefficients methods) and direct effects from the product approach is shown. In the product approach, exposure-to-mediator effects are multiplied with mediator-to-outcome direct effects and summed up across mediators to get the indirect effect. The direct effect is then calculated by subtracting this indirect effect from the total effect. The right panel shows the agreement between total effects obtained from the univariable MR regression and total effects reconstructed by summing the direct and indirect effects derived from the MVMR regressions. In the latter, the direct effect refers to the "Direct effect – MVMR" from the left panel and the indirect effect

to the one obtained in the product approach. The *R* coefficient displayed in the plot area is the Pearson correlation coefficient (identity line is plotted in blue). Results are shown for all 2,069 DNAm-trait pairs colour-coded by the physiological category of the trait as defined in Supplementary Fig. 8.

Multi-omics mechanisms of action

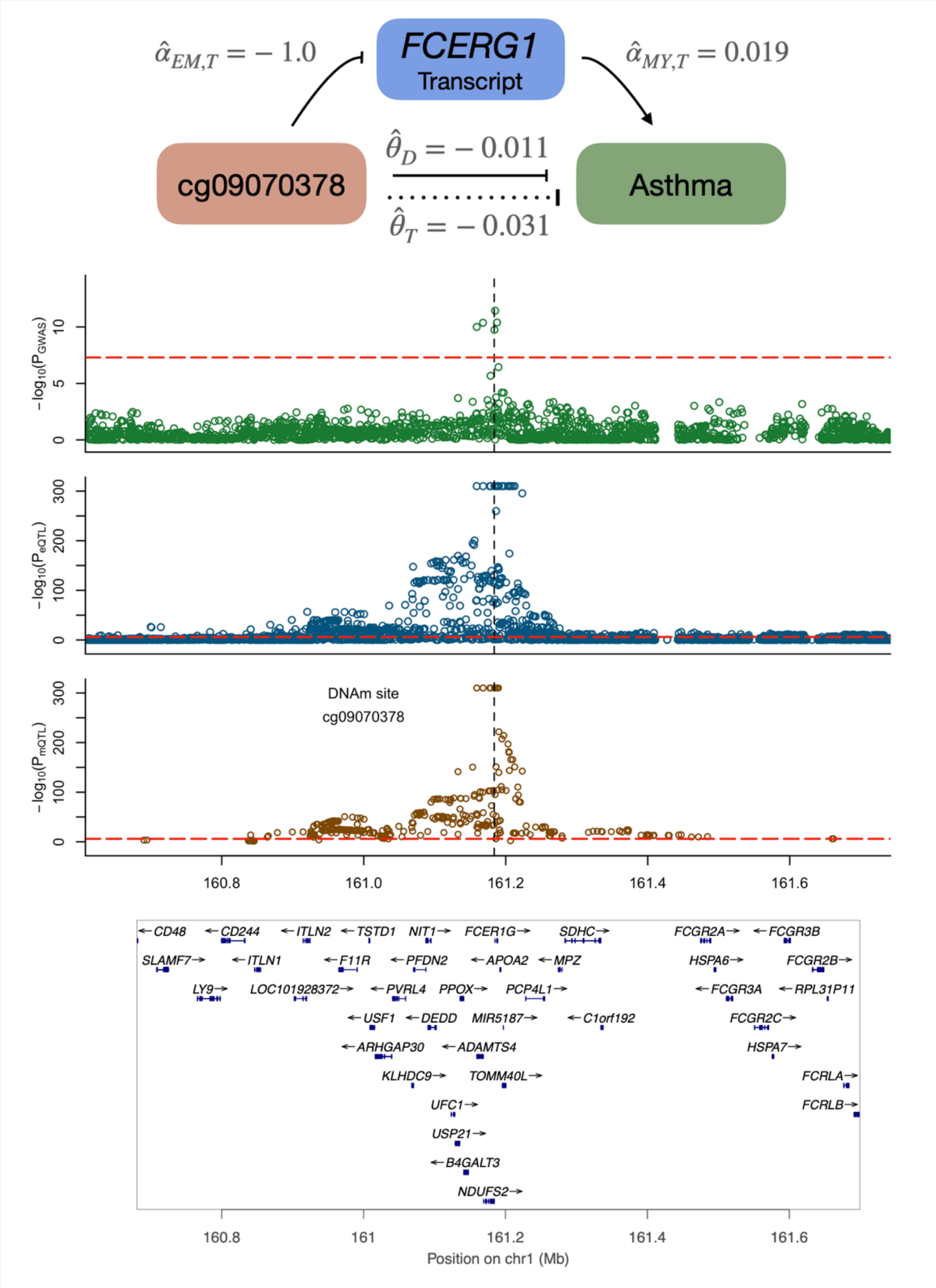

Supplementary Figure 21. Plausible DNAm-transcript-trait regulatory mechanism for asthma disease at the *FCERG1* locus. The top row displays a schematic of the mechanism with the calculated univariable and multivariable MR effects. The three following rows show the regional SNP associations (-$\log_{10}$(p-values)) with the trait (green), transcript (blue) and DNAm probe (brown), respectively. Red dashed lines indicate the significance thresholds of the respective SNP associations and the vertical black dashed line represents the DNAm probe position. The bottom row shows the positions of the genes in the locus with their respective strand direction.

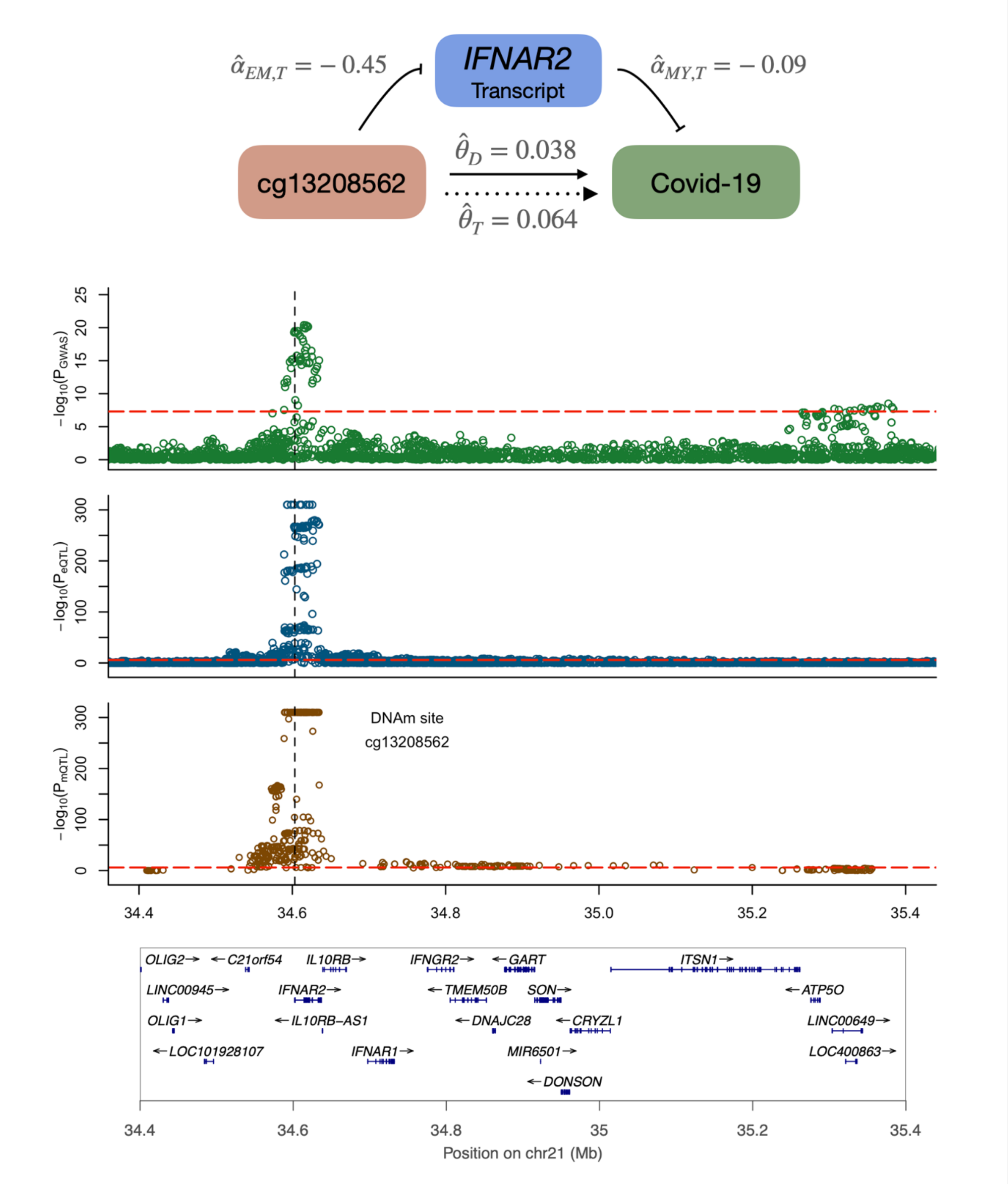

Supplementary Figure 22. Plausible DNAm-transcript-trait regulatory mechanism for Covid-19 (hospitalized vs population) at the *IFNAR2* locus. Same figure composition as Supplementary Fig. 21.

Supplementary Tables

Supplementary Table 1. Means and interquartile ranges of the simulation parameters as observed in real data. The full distribution of each parameter is shown in Supplementary Fig. 5.

	Quartile 1	Median	Mean	Quartile 3
$N_{\text{med,pot}}$	7	12	14.7	21
$N_{\text{med,sig}}$	1	2	3.3	4
m_E	3	4	5.09	6
m_M	1	3	5.65	8
h^2_E	0.179	0.319	0.403	0.539
$h^2_{M,\text{direct}}$	4.75E-03	0.0148	0.0418	0.047
var(α_{EM})	5.48E-03	0.0196	0.0789	0.0751
var(α_{MY})	1.18E-04	7.37E-04	9.52E-03	3.78E-03
ρ	-0.39	-0.0216	-0.0112	0.361

Supplementary Table 2. Values used in the different simulation settings to mimic mediation of DNAm-to-trait effects through transcript levels. Results of the default model are shown in Supplementary Fig. 6, results of varying the sample size N_M, the mediator selection threshold P_{EM}, and heritabilities $h^2_{M,\text{direct}}$ and h^2_E in Fig. 2, and the remaining simulation settings in Supplementary Fig. 7. Median parameter values are used in the default model and values comprising the interquartile range when varying the respective parameter.

<table>
<tr><th></th><th>Default model (median values)</th><th>Varying N_M</th><th>Varying P_{EM}</th><th>Varying $h^2_{M,\text{direct}}$</th><th>Varying h^2_E</th><th>Varying m_E</th><th>Varying $N_{\text{med,sig}}$</th></tr>
<tr><td>N_{med}</td><td colspan="2">12</td><td>20</td><td colspan="3">12</td><td>20</td></tr>
<tr><td>$N_{\text{med,sig}}$</td><td colspan="6">2</td><td>[1 - 10]</td></tr>
<tr><td>m_E</td><td colspan="5">4</td><td>[3 - 12]</td><td>4</td></tr>
<tr><td>m_M</td><td colspan="7">3</td></tr>
<tr><td>h^2_E</td><td colspan="4">0.319</td><td>[0.05 - 1]</td><td>0.1, 0.3, 0.5</td><td>0.319</td></tr>
<tr><td>$h^2_{M,\text{direct}}$</td><td colspan="3">0.0148</td><td>[3E-04 - 0.64]</td><td colspan="2">0.0148</td><td>4.75E-03, 0.0148, 0.047</td></tr>
<tr><td>ρ</td><td colspan="7">-0.02</td></tr>
<tr><td>P_{EM}</td><td colspan="2">0.01</td><td>[1E-06 - 1]</td><td colspan="4">0.01</td></tr>
<tr><td>N_M</td><td>30,000</td><td>[100-100,000]</td><td colspan="5">30,000</td></tr>
<tr><td>N_E</td><td colspan="7">30,000</td></tr>
<tr><td>N_Y</td><td colspan="7">300,000</td></tr>
</table>

**var(α_{EM})**	0.02
**var(α_{MY})**	1.00E-03
**MP**	0.35

Supplementary Table 3. Enrichment analysis of negative DNAm-to-transcript causal effects within each annotation group. The first two columns show the number of distinct DNAm-transcript pairs with negative and positive causal effects, respectively. An enrichment analysis for negative causal effects was conducted as a two-sided Fisher's test where each annotation group was tested against the remaining other groups combined. Annotation groups significantly enriched or deprived for negative causal effects (after correcting for multiple testing at $P < 0.05/6$) are highlighted in bold.

Annotation group	DNAm → Transcript negative effect	DNAm → Transcript positive effect	Proportion of negative effects	OR (negative effect enrichment)	P-value (negative effect enrichment)
**1stExon**	291	188	0.608	**1.33**	**2.67E-03**
3'UTR	863	828	0.510	0.89	1.63E-02
5'UTR	1773	1429	0.554	1.07	8.03E-02
**Body**	9675	8824	0.523	**0.87**	**2.15E-10**
**TSS1500**	4380	3433	0.561	**1.12**	**1.24E-05**
**TSS200**	1702	1284	0.570	**1.15**	**3.81E-04**
