# Supplementary material for: Quantifying the role of transcript levels in mediating DNA methylation effects on complex traits and diseases
Source: Nat Commun. 2022 Dec 7;13:7559. doi: 10.1038/s41467-022-35196-3 (PMC9729239; doi:10.1038/s41467-022-35196-3)
Supplement: Supplementary file 3 — Description of Additional Supplementary Files [file 41467_2022_35196_MOESM3_ESM.pdf]

Description of Additional Supplementary Files

File Name: Supplementary Data 1
Description: List of GWAS summary statistics corresponding to the outcome traits that were evaluated in the MVMR mediation analyses.

File Name: Supplementary Data 2
Description: Putative DNAm-to-complex traits mechanisms of action through transcript levels.
