# Supplementary material for: Quantifying the role of transcript levels in mediating DNA methylation effects on complex traits and diseases
Source: Nat Commun. 2022 Dec 7;13:7559. doi: 10.1038/s41467-022-35196-3 (PMC9729239; doi:10.1038/s41467-022-35196-3)
Supplement: Supplementary file 6 — Reporting Summary [file 41467_2022_35196_MOESM6_ESM.pdf]

nature portfolio

Corresponding author(s): Marie C Sadler, Zoltán Kutalik

Last updated by author(s): Nov 15, 2022

Reporting Summary

Nature Portfolio wishes to improve the reproducibility of the work that we publish. This form provides structure for consistency and transparency in reporting. For further information on Nature Portfolio policies, see our Editorial Policies and the Editorial Policy Checklist.

Statistics

For all statistical analyses, confirm that the following items are present in the figure legend, table legend, main text, or Methods section.

n/a	Confirmed	
☐	☒	The exact sample size (*n*) for each experimental group/condition, given as a discrete number and unit of measurement
☒	☐	A statement on whether measurements were taken from distinct samples or whether the same sample was measured repeatedly
☐	☒	The statistical test(s) used AND whether they are one- or two-sided<br>*Only common tests should be described solely by name; describe more complex techniques in the Methods section.*
☐	☒	A description of all covariates tested
☐	☒	A description of any assumptions or corrections, such as tests of normality and adjustment for multiple comparisons
☐	☒	A full description of the statistical parameters including central tendency (e.g. means) or other basic estimates (e.g. regression coefficient) AND variation (e.g. standard deviation) or associated estimates of uncertainty (e.g. confidence intervals)
☐	☒	For null hypothesis testing, the test statistic (e.g. *F*, *t*, *r*) with confidence intervals, effect sizes, degrees of freedom and *P* value noted<br>*Give P values as exact values whenever suitable.*
☒	☐	For Bayesian analysis, information on the choice of priors and Markov chain Monte Carlo settings
☐	☒	For hierarchical and complex designs, identification of the appropriate level for tests and full reporting of outcomes
☐	☒	Estimates of effect sizes (e.g. Cohen's *d*, Pearson's *r*), indicating how they were calculated

Our web collection on statistics for biologists contains articles on many of the points above.

Software and code

Policy information about availability of computer code

Data collection	In this paper no software was used for data collection.
Data analysis	Software (https://github.com/masadler/smrivw, https://doi.org/10.5281/zenodo.7324709) and detailed documentation (https://github.com/masadler/smrivw/wiki), including an example on real data, is provided to repeat and conduct univariable and multivariable omics Mendelian randomization analyses. This software is based on the SMR-software v1.03 (https://cnsgenomics.com/software/smr). Heterogeneity Q-statistics were computed based on code from the TwoSampleMR package (v0.5.6, IVW-method). DNAm probe annotations with respect to the assessed transcript were obtained using the IlluminaHumanMethylation450kanno.ilmn12.hg19 R package (v0.6.1).

For manuscripts utilizing custom algorithms or software that are central to the research but not yet described in published literature, software must be made available to editors and reviewers. We strongly encourage code deposition in a community repository (e.g. GitHub). See the Nature Portfolio guidelines for submitting code & software for further information.

Data

Policy information about availability of data

All manuscripts must include a data availability statement. This statement should provide the following information, where applicable:

- Accession codes, unique identifiers, or web links for publicly available datasets
- A description of any restrictions on data availability
- For clinical datasets or third party data, please ensure that the statement adheres to our policy

Methylation QTLs used in this study are from the GoDMC mQTL meta-analysis and are available on the GoDMC Consortium website (http://mqtldb.godmc.org.uk/downloads). Expression QTLs are from the eQTLGen eQTL meta-analysis and are available on the eQTLGen Consortium website (https://www.eqtlgen.org/cis-

eqtls.html). The list of GWAS summary statistics used in this study is in Supplementary Data 1, all of which are publicly available. UK10K individual-level data are available upon request (https://www.uk10k.org/data_access.html). Source data are provided with this paper.

Field-specific reporting

Please select the one below that is the best fit for your research. If you are not sure, read the appropriate sections before making your selection.

- [x] Life sciences
- [ ] Behavioural & social sciences
- [ ] Ecological, evolutionary & environmental sciences

For a reference copy of the document with all sections, see nature.com/documents/nr-reporting-summary-flat.pdf

Life sciences study design

All studies must disclose on these points even when the disclosure is negative.

Sample size	mQTL data are from a meta-analysis (GoDMC consortium) including up to 32,851 individuals. eQTL data are from a meta-analysis (eQTLGen consortium) including up to 31,684 individuals. GWAS summary statistics include sample sizes > 100,000 with the average sample size being about 320,000. Sample sizes were reported for each GWAS summary statistics and used as needed. For case-control GWAS summary statistics, the effective sample size (N_eff) was calculated as N_eff = (4*Ncase*Ncontrol)/(Ncase + Ncontrol). mQTL and eQTL datasets are the largest publicly available datasets of their kind. Likewise, for each outcome trait, we used the largest GWAS summary statistics, publicly available, at the time of analysis.
Data exclusions	No data was excluded from mQTL and eQTL datasets. If the GWAS analysis provided meta-analysis separate for European and non-European ancestries, we chose the summary statistics restricted to European ancestry to match our mQTL and eQTL datasets.
Replication	Simulation studies were replicated 500 times for each setting that was tested. For the real data application, no sufficiently large other datasets are available for replication. However, we performed a range of sensitivity analyses to assess the robustness of the results.
Randomization	There was no group assignment performed in this study since it was not experimental.
Blinding	Blinding is not relevant to our study since it was not experimental. Data collection and processing were done in previous studies.

Reporting for specific materials, systems and methods

We require information from authors about some types of materials, experimental systems and methods used in many studies. Here, indicate whether each material, system or method listed is relevant to your study. If you are not sure if a list item applies to your research, read the appropriate section before selecting a response.

Materials & experimental systems

n/a	Involved in the study	
☒	☐	Antibodies
☒	☐	Eukaryotic cell lines
☒	☐	Palaeontology and archaeology
☒	☐	Animals and other organisms
☒	☐	Human research participants
☒	☐	Clinical data
☒	☐	Dual use research of concern

Methods

n/a	Involved in the study	
☒	☐	ChIP-seq
☒	☐	Flow cytometry
☒	☐	MRI-based neuroimaging
